# Characterization and factors associated with diarrhoeal diseases caused by enteric bacterial pathogens among children aged five years and below attending Igembe District Hospital, Kenya

**DOI:** 10.11604/pamj.2013.16.37.2947

**Published:** 2013-10-04

**Authors:** Shirley Karambu, Viviene Matiru, Michael Kiptoo, Joseph Oundo

**Affiliations:** 1 Field epidemiology and Laboratory training programme, Kenya; 2 Ministry of Public Health and Sanitation, Kenya; 3 Jomo Kenyatta University of Agriculture and Technology, Kenya; 4 Kenya Medical Research Institute, Kenya; 5 United States Army Medical Research Unit – Kenya

**Keywords:** Enteric bacterial pathogens, diarrhea, children five, Igembe District Hospital, Kenya

## Abstract

**Introduction:**

Diarrhoea remains a major public health problem in East African nations such as Kenya. Surveillance for a broad range of enteric pathogens is necessary to accurately predict the frequency of pathogens and potential changes in antibiotic resistance patterns.

**Methods:**

A cross sectional study was conducted in Igembe District Hospital in Meru County to determine the burden and factors associated enteric bacterial infection among children aged five years and below. Stool samples were collected between March and July 2012. Bacterial pathogens were identified and antibiotic susceptibility of bacterial isolates was ascertained. Questionnaire was administered to the 308 study participants to identify the modifiable risk factors. Data was entered and analyzed using *Epi Info* version 3.5.3.

**Results:**

The study recruited 308 children. The mean age was 27.25 months, median of 26.0 months and age range between 2-60 months. The bacterial isolation rates were ETEC 9.1%, EPEC 6.8% and EAEC 12.3%, Salmonella paratyphoid (10.4%), *Shigella flexineri* (1.9%) and *Shigella dysentriae* (0.9%). Over 95%, of the isolates were resistance to amoxicillin, sulphinatozole, cotrimoxazole. Six factors were independently associated with diarrhoeal diseases, occupation of the parent/ guardian (miraa business) (OR=1.8, CI:1.44-4.99),care taker not washing hands after changing napkins (OR= 1.6, CI:1.2-19.7), child drank untreated water from the river (OR= 2.7, CI:2.4-9.9) child not exclusively breastfed (OR= 2.4, CI:2.1-10.5),child did not Wash hands before eating (OR=2.2, CI:1.91-16.3) and after visiting toilet (OR=3.7,CI:2.8-39.4). Eating of mangoes was found to be protective against diarrhoea (OR=0.5, CI:0.03-0.89).

**Conclusion:**

The bacterial pathogens were found to be a significant cause of diarrhoea in the study participants. We established higher resistance to several commonly prescribed antibiotics. Several factors were significantly association with diarrhoea illness. We recommend multifaceted approach that acknowledges the public health aspects that would reduce the burdenof diarrhoea infectious as identified in this study.

## Introduction

Diarrhoea is the passage of three or more loose or liquid stools per day or more frequent passage than is normal for the individual [1]. It is usually a symptom of gastrointestinal infection diarrhoea diseases due to infection constitute a major burden of disease [2]. *Enterotoxigenic E. coli, Salmonella paratyphi, Shigella spices* and virus appeared to be the most common etiological agents but Certain circumstances are associated with and especially high incidence of acute diarrhoeal disease [3] however, the causes of approximately 40% of the cases are still unknown [4]. Diarrhoea is a major cause of childhood disease in the developing world, global mortality estimates from diarrhoea and its complications range from 1.5 to 5.1 million deaths per year for children under the age of five [5]. Diarrhoea is the second leading killer of children, and nearly one in five children under the age of five dies as a result of dehydration, weakened immunity or malnutrition associated with diarrhoea [6]. Of the estimated total 10.6 million deaths among children younger than five years of age worldwide, 42 percent occur in the world health organization (WHO) African region [7]. Although mortality rates among these children have declined globally from 146 per 1,000 in 1970 to 79 per 1,000 in 2003 [8] the situation in Africa is strikingly different compared with other regions of the world African region shows the smallest reductions in mortality rates and the most marked slowing down trend.

The under-five mortality rate in the African region is seven times higher than that in the European region. In 1980 this difference was equal to 4.3 times [9].

During the 1990s, the decline of under-five mortality rates in 29 countries of the world stagnated, and in 14 countries rates went down but then increased again. Most of these countries are from the African region. One possible contributing factor that may contribute to this situation is the human immunodeficiency virus/acquired immune deficiency syndrome (HIV/AIDS) epidemic in the region, but an underlying weakness of the implementation capacity of the health system is also likely to blame[10]. Dehydration resulting from diarrhoea can be fatal and causes approximately 1.8 million deaths every year [2]. Of all medical conditions, diarrhoea is the second leading cause of healthy time lost to illness. Around 90% of all diarrhoea-related deaths occur in children under five years of age living in LMIC. Similarly to all-cause mortality, global estimates of the number of deaths due to diarrhoea have shown a steady decline, from 4.6 million in the 1980s,1982 [11] to 3.3 million in the 1990s [5] to 2.5 million in the year 2000[12]. 21.9% of all deaths of children up to 4yrs of age in sub-Saharan Africa in the year 2000 were due to diarrhoea corresponding to 935,000 deaths attributable to diarrhoea [13]. It has been estimated that 88% of all diarrhoeal diseases are as a result of contaminated water and inadequate hygiene and sanitation [14].

Lack of access to safe water and adequate sanitation facilities is a serious problem worldwide. Approximately 884 million people lack access to improved water sources and 2.6 billion people do not have access to improved sanitation facilities [15], this leads to open defecation and the improper disposal of feces. It is estimated that in developing countries, 25% of people defecate in the open [16]. Microbiological culture and microscopy remain the standard, despite their limited sensitivity [17]. Newer immunological and nucleic acid-based tests to detect pathogen-specific factors hold great promise for all diarrhoea agents, but they are too expensive or require specialized instrumentation and trained technicians [18]. The WHO treatment recommendations for diarrhoeal illness include the use of oral rehydration therapy to replenish lost fluids and electrolytes, continued feeding as the child experiences diarrhoea, and administration of zinc for 10-14 days (10mg for 6 months). Diarrhoeal diseases remain among the five top preventable killers of children underfive in developing countries [19]. Improving domestic hygiene practices is potentially one of the most effective means of reducing the global burden of disease in children [20]. We conducted this study to Characterization and factors associated with diarrhoeal diseases caused by enteric bacterial pathogens among children aged five years and below attending Igembe District Hospital, Kenya.

## Methods

### Study site

The study was carried out in Igembe District Hospital. The Hospital is located in Maua town about 50km from Meru town in Meru County. Igembe south district covers an area of approximately 270.70 Km2 and has an estimated population of 145,301[21]. The district is home to a large Somali population whose main occupation is trading in Khat (*Cartha edulis*) in the study area. The climate of the area is cold with two distinct seasons, warm and rainy. The site was conveniently selected because it is the district referral health hospital.

### Study design

We conducted a laboratory based cross sectional study that targeted all children aged ≥ 5 years presenting with diarrhoea recruited from the maternal and child health clinic (MCH). We defined any diarrhoea as the passage of three or more liquid stools within 24 hours, or any number (more than three times) of loose stools accompanied with mucous within 24 hours as per WHO definition.

### Experimental procedure

We collected data on social and demographic factors, immunization status, breast feeding and environmental risk factors using fully structured questionnaire. The study was approved by Kenya Medical Research Institute (KEMRI) ethical review committee. Written informed consent was obtained from interviewees/participants parents / guardian after complete explanation of the study content and purpose.

A minimum sample size of 308 study participants was calculated and enrolled in the study using systematic random sampling. Stool samples were collected on the same day the child arrived at the hospital with episodes of diarrhoea. Identification of bacterial pathogens and antimicrobial susceptibility was conducted. Social demographic and modifiable factors were analysed.

### Data Analysis

Data was analysed using Epi Info version 3.5.3 (CDC Atlanta, USA). The Frequencies and proportions were described and the exposure and risk factors determined by univariate analysis while multivariate analysis was done to come up with the best fit model of the of the factors associated.

## Results

The study enrolled 308 participants (children) 5years and below seeking treatment at Igembe District Hospital from March - July, 2012. Majority (74.7%, n= 230) came from Igembe south district. Female children constituted 55.2 percent (n=170).

The ratio of female to male being (1.2:1) as shown in Table 1.

**Table 1:** Socio-demographic Characteristics of Study Participants

Descriptive Variables	Frequency (N=308)	Percent (%)
**Age group of the study participants**		
>12 months	54	17.5
12-23 months	82	26.6
24-35 months	61	19.8
36-47 months	67	21.8
48- 60 months	44	14.3
**Sex of the study participants**		
Male	138	44.8
Female	170	55.2
**Order of birth of the study participants**		
1	36	11.7
2	86	27.9
3	76	24.7
4	49	15.9
5	31	10.1
6	16	5.2
7	9	2.9
8	5	1.6
**Relationship to the study participants (child)**		
Caretaker	10	3.2
Father	48	15.6
Mother	228	74.0
Others	22	7.1
**Level of education of the parents / guardians of the study participants**		
None	64	20.8
Primary	92	29.9
Secondary	103	33.4
Tertiary	49	15.9
**Occupation of the parents / guardians of the study participants**		
Business	122	39.6
Employment	68	22.1
Farmer	65	21.1
Housewife	53	17.2
**Religion of the study participants**		
Christian	261	84.7
Islam	33	10.8
Others	14	4.5

All the participants were 60 months and below ranging from 2-60 months. The Mean age of the participants was 27.25 months, with a Median of 26 months, Mode of 36 months and a Standard Deviation of 15.8. The age group between 12-23 months had the majority of the participants (n=82, 26.6%) The prevalence rate was lowest in over 4year-old implying that children aged between 12 months and 48 months were more susceptible to diarrhoeal than those >4yrs of age (Table 1). The main caretakers of the children were their parents (mothers 74%, fathers 15.6%). Majority of these mothers had secondary level of education (33.4%) and most of the parents were business persons (Table 1).

The Bivariate analysis was performed to determine the possible risks or protective factors for diarrhoea in the study participants. The following factors were found to have likely caused diarrhoea; occupational of the study participants parents (OR=2.7, CI:2.91-16.93), children not washing hands before eating and after visiting the toilet (OR= 4. 01, CI:3.89-48.92), (OR= 2.8, CI: 2.71-6.96) respectively. Care taker not washing hands after changing napkins (OR= 2.1, CI: 1.84-4.98) the children who drunk untreated water (OR =1.6, CI: 1. 09 - 3.71), children who were not exclusively breast feed (OR =3.4, CI:1.65-27.79) and children who eat mangoes (OR=1.4, CI: 1.01-4.33). The following were less likely to have caused diarrhoea in the study participants; sex of the study participants (OR= 0.6, CI: 0.63-0.89),children drinking water from the river (OR= 0.89, CI: 0.57-0.95) Children who never used toilet regularly (OR= 0.2,CI: 0.1-0.68), children who do not wash their hands after eating ( OR= 0.7,CI: 0.33-0.99) and children with parents with formal education (OR= 0.43, CI: 0.15-10.51).

Table 2 shows results of bivariate analysis. Multivariate analysis of the variables was done to control for multiple potential confounders and the following variables were independently found to be risk factors;Occupation of the parent/ guardian ( khat business) (AOR=1.8, CI: 1.44-4.99),Care taker not washing hands after changing napkins (AOR= 1.6, CI: 1.2-19.7), child drank untreated water from the river (AOR= 2.7, CI: 2.4-9.9) child not exclusively breastfed (AOR= 2.4, CI:2.1-10.5) child did not Wash hands before eating (AOR=2.2, CI: 1.91-16.3) and after visiting toilet AOR=3.7,CI:2.8-39.4). Eating of mangoes was found to be protective factors against diarrhoea (AOR=0.5, CI: 0.03-0.89) (Table 3).

**Table 2:** Bivariate analysis of factors associated with diarrhea among the study participants in Igembe district hospital, Kenya, 2012

Factors	OR	95% CL	p.v
occupation of the parents of the participants	2.9	(2.71 -16.93)	0.025
child not washing hands before eating	4.01	(3.89 -48.9)	0.015
child not washing hands after visiting toilet	2.8	(2.71 -6.96)	0.011
care taker not washing hands after changing napkin	2.1	(1.84 - 4.98)	0.003
child drunk untreated	1.6	(1.09 - 3.71)	0.077
child not exclusively breast feed	3.4	(1.65 - 27.79)	0.005
child eat mangoes	1.4	(1.01 - 4.33)	0.018
child drunk water from the river	0.89	(0.57 - 0.99)	0.023
child sex	0.6	(0.52 - 0.89)	0.006
child who used toilet regularly	0.2	(0.1 - 0.68)	0.003
child wash hands after eating	0.7	(0.33 - 7.9)	0.004
child with parent with formal education	0.43	(0.15 - 10.51	0.571

No - number,%- percent, CI- confidence interval, OR- odds ratio, - LL- lower limit, UL- upper limit.

**Table 3:** Multivariate Analysisof Risk factors associated with diarrheal diseases among the study participants in Igembe district hospital, Kenya in 2012

Factors	95% CI			
Demographic factors	AOR	LL	UL	p.v
Occupation of the parents /guardian (miraa business)	1.8	1.44	4.99	0.013
**Hand washing**				
Child did not wash hands before eating	2.2	1.91	16.3	0.02
Child did not wash hands after visiting toilet	3.7	2.8	39.4	0.014
Care taker did not wash hands after changing napkin	1.6	1.2	19.7	0.011
**Exposure**				
Child drunk untreated water	2.7	2.4	9.9	0.02
Child not exclusively breastfeed	2.4	2.1	10.5	0.01
Child eat mangoes	0.5	0.03	0.89	0.03

**Key:**% - percent, AOR- Adjusted odds ratio, LL - lower limit, UL- upper limit, CI- confidence interval.

## Discussion

The study was to characterize the diarrhoea diseases caused by enteric bacterial pathogens and the factors associated. Three enteric bacteria were isolated as the cause of the diarrhoeal disease with an isolation rate of (56.2%). A study done in Tanzania by Gascon et al., [22] had almost the same isolation rate (52.2%). The spices and pathotypes of the same isolates could closely be compared. *Escherichia coli* (28.2%) ETEC (9.1%), EPEC (6.8%) andEAEC (12.3%), *Salmonella paratyphi* (10.4%), Shigella species (2.9%), Shigella flexineri and Shigella dysenteriae (0.65%, 2.3%) respectively [22]. The findings concur with several other similar studies demonstrating that Salmonella, *Campylobacter, Escherichia and Shigella* are the most common types of bacterial infections causing diarrhoea in Western societies [22]. A study done in Kenya in 2008 established that EAEC (8.6%), (ETEC 7.9%). EPEC (7.4%), Shigella species (4.1%) and Salmonella species (3.9%) were the most frequently identified bacterial pathogenic agents isolated [24]. A similar study done in Kenya (Mbagathi District Hospital) found that *Escherichia coli* (93.83%) was the most commonly isolated pathogen followed by Salmonella 3.7% and Shigella 2.4%) [25].

The findings in this study concur with a similar study done in Kericho and Kisumu hospitals in Kenya, showing that most of the isolates were diarrhoeagenic Escherichia coli and Salmonella spices [26]. A study done in Central Africa gave an isolation rate of 12.1% of EPEC almost double the findings of this study 6.8% [27]. Our isolation rate was 56.2%, this can be closely compared with a similar study done in Northern Jordan that yielded enteropathogens rate of 66.4%. Nevertheless Escherichia coli isolates in the same study isolated less than twice our findings (12.8%). Other isolates in the same study were as follows; EAEC 10.2%, ETEC 5.7%, Shigella species 4.9%, and Salmonella species 4.5% [28]. These findings can closely be compaired with the findings from this study (11.7%, 9.7%, 2.9%, and 10.4% respectively. Studies done in Argentina and Bangladesh found that Rotavirus, Shigella species, and ETEC were the most common isolates from diarrhoeal samples [29]. Three commensals were isolated from our study but at a very low percentage. Progression in understanding the relation between commensal bacteria and human health is likely to promote the identification of new approaches to disease prevention and treatment. Numerous reports have described the isolation of Aeromonas from patients with acute diarrhoea, but the bacterium can also be isolated from stool of healthy persons [30]. In a study done in Turkey, numerical taxonomy of Aeromonasstrains isolated from different sources revealed the presence of potentially pathogenic *Aeromonas* species especially in food [31]. The vast majority of *Klebsiella* infections, however, are associated with hospitalization as opportunistic pathogens [32] and considered as commensal in this study. Proteus species infections occur worldwide and are part of the human intestinal flora but can cause infection upon leaving this location [33]. We observed higher resistance in amoxicillin, cotrimoxazole, streptomycin and tetracycline (99%, 96%, 97%, 81%) respectively. This corresponds with a study done in Kenya that found higher resistance in some similar drugs e.g. tetracycline (81%) [34]. Similar resistance was experienced in amoxicillin and cephalexin. This was revealed in a study done in Abuja, Nigeria [35]. None of the bacterial pathogens isolated showed resistance to Nalidixic acid, ciprofloxacin, kanamycin and ofloxacin. We related this high resistance of some particular drugs with numerous private clinics and chemists not controlled in the area leading to purchases of antibiotics over the counter from unqualified drug sellers with several drug alternatives from the prescribed drugs [36]. We also associated the resistance of some drugs with imprudent use of antibiotics in the area purchased without prescription from the private chemists as evidenced by the 48.4% (n= 149) of the children who sought treatment before attending the hospital with 21% (n= 32) having been treated with different antibiotics purchased from the private chemists.

A number of antibiotics can cause diarrhoea in both children and adults and the diarrhoea is usually mild and typically does not cause dehydration or weight loss [37]. In this study we found that more than thirty percent of the study participants were treated with antibiotics from the hospital whose susceptibility profile is not determined. Acute diarrhoea was experienced in two hundred and seventy seven children (about 90%), with only twenty two participants having persistent diarrhoea (7%). Irrational antibiotic use during acute diarrhoeal episodes (p38]. A different study revealed that underweight children had a higher risk of diarrhoea becoming persistent [39]. Two different studies done in Bangladesh [40] and Zaire [41] revealed that persistent diarrhoea accounted for 5.8% of the 1916 diarrhoea episodes and persistent diarrhoea associated with malnutrition was responsible for nearly half of the diarrhoea deaths. This can closely be compared with the findingsof this study where we found only 7% participants had persistent diarrhoea. The odd of diarrhoea was not significantly associated with sex (female 55.2% and male 44.8%). This agrees with a study done in teaching hospital in Nigeria where males affected were 54% and female 40%, with the odds having diarrhoea not significantly related to sex [42]. A study done on the children living in urban slums in Salvador, Brazil on incidence of diarrhoea revealed that male children were associated with episodes of diarrhoea as compaired to their female counterparts [43]. A similar study done in Denmark found that being a male, one stands a chance of getting diarrhoea [44]. These two studies contradict our finding in this study. Our finding was also contradicted by a similar study done in India in a pediatric ward of Command Hospital Pune where Boys were more likely to be afflicted with a diarrhoeal disease than girls (63.16% vs. 36.84%) [45]. Schilling did a similar study in Kenya and found no association between gender and diarrhoea disease [25]. A different study done in Guatemala found that there existed no significant association between males and females aged 0-17 months old although males of 18 months and older experienced high number of episodes of persistent diarrhoea than females in the same age group [46]. We found higher prevalence of diarrhoea in age group 12-23 months (26.6%), this coincided with a study done in Kathmandu, Nepal that revealed that a higher prevalence of diarrhoea was found in age groups less than 2 years [47]. However a study done in Abuja, Nigeria publicized a higher prevalence of diarrhoea in children in the age group 7-12 months [48] contradicting our report in this study. According to a study done in Saudi Arabia by Mazrous and others, Incidence of diarrhoea was significantly associated with fathers′ occupation (p=0.0011) but no significant association was found between the incidence of diarrhoea and parents′ education [36]. This agrees with our findings, we establishing that diarrhoea was significantly associated with the occupation of the parent/guardian (OR= 1.8, CI 1.44-4.99) but no significant association was found between the incidence of diarrhoea and parents′ level of education. The study established that the community experiences a lot of social-economic challenges evidenced by the occupation of the parents’/guardian where only 22% (n=68) are formally employed.

The household size was high with almost half (49.7%, n=153) of the participants having over nine members, with no significant association found between the incidence of diarrhoea and household size.our finding was opposed by a similar study done in India, pediatric wards of Command Hospital Pune that found a family size of more than 4 was associated with a higher incidence of diarrhoea than larger family sizes (p v= 0.05) [49]. The same study established that diarrhoea was more common in overcrowded households than in non-crowded households (p v=0.05). Immunization is a corner stone of public health in eradicating the immunizable diseases in many regions of the world. Immunization has been seen as one of the most cost effective public health interventions in most regions of the world. In our study 89%, n=273 of participants had received some immunization and out of 273, 56.5% had some pathogen isolated in their stool sample, though no association was found with diarrhoea.

Several Studies have shown the beneficial effects of breast-feeding in preventing morbidity and mortality from diarrhoea in infants. A case-control study in Brazil has shown that young infants who are not breast-fed have a 25-times greater risk of dying of diarrhoea than those who are exclusively breast-fed. We found that the children who were not exclusively breast-fed had 2.4 times greater (OR=2.4, CI 2.1-10.5) risk of getting diarrhoea than those who were exclusively breast-fed. A longitudinal study in the urban slums of Lima, Peru found that exclusively breast-fed infants had a reduced risk of diarrhoeal morbidity when compared with infants receiving only water in addition to breast-milk [50]. Infants who were partially breastfed or not breastfed had a risk of diarrhoeal death 3.94 times greater (95% CI: 1.47-10.57) than exclusively breastfed infants. According to Baker and others, an episode of diarrhoea was significantly less likely to last for six or more days if an infant was breastfed for three or more months. The risk of developing diarrhoea increases as the amount of breast milk an infant receives decreases. Studies have compared exclusively breastfed infants, to infants who were exclusively formula-fed had an 80% increase in their risk of developing diarrhoea in the first year of life the incidence of diarrhoeal illnesses among breastfed infants was half that of formula-fed infants [33, 41, 50]. All these study findings concur with the findings of this study that reveals a significant association of diarrhoea with children who were not exclusively breast feed (OR= 3.4, CI:2.1-10.5). Lack of exclusive breast-feeding up to the first four months of life (p 50]. These studies, along with numerous others in developing countries, point to the need to extend the duration of exclusive breast-feeding to at least 4-6 months [50]. Water sources was one of the main risk factors associated with diarrhoea in children less than 5 years this was according to a study done in Zambia [49, 50]. In a different study use of unsafe drinking water (p33, 41, 50]. Treatment of water before drinking was done by about half of the study participants (50.9% n=157) with majority of participants identifying with boiling method (79.6%) however none of the method was found to have any association with diarrhoea. Children who washed hands before eating are about four times less likely to get diarrhoea infection as compared to those who did not wash their hands and about three times less likely to those who wash hands after visiting toilet (OR= 4. 01, CI:3.89-48.92), (OR= 2.8, CI: 2.71-6.96) respectively. The same opinion was found by Cochrane review of Database Systematic that found out that hand washing reduced episodes of diarrhoea by 30%, this intervention has been shown to reduce transmission of diarrhoea-causing organisms [7, 10, 50]. These findings are in harmony with a study done on a systematic and meta-analysis of sanitation, water and hygiene in less developed countries, expounding that washing one's hands with soup is another important barrier to transmission. A study done in India showed clear evidence of 54% decrease in microbial flora after hand washing [50]. Hand washing promotion and interventions are estimated to have the potential to prevent one million deaths from diarrhoeal diseases. In our study we found that care taker not washing hands after changing napkin can expose the child to diarrhoea illness more than one and half times as compared to those washing hands after changing the napkins (OR=1.6, CI - 1.2-19.7).

## Conclusion

The bacterial pathogens were found to be a significant cause of diarrhoea in children five years and below in Igembe district Hospital. We established higher resistance to several commonly prescribed antibiotics and this was associated with irrational use of antibiotic for the treatment of acute diarrhoea. Significant association of diarrhoea was found in children who were not exclusively breast feed, whose who drank untreated water from any source. Children who never washed their hands before eating, after visiting toilet, those care takers did not wash their hands after changing napkins and children whose parents/ guardian occupation was in khat business. We recommends Multifaceted approach that acknowledges the public good aspects of health situation that may be important in reducing the burden of infection of diarrhoea as identified as risk factors in this study, through recommendation of more health education regarding appropriate health seeking and greater interventions at the community level by engaging community health workers in diarrhoea prevention, control and treatment.
